# Potentially Preventable Deaths from the Five Leading Causes of Death — United States, 2008–2010

**Published:** 2014-05-02

**Authors:** Paula W. Yoon, Brigham Bastian, Robert N. Anderson, Janet L. Collins, Harold W. Jaffe

**Affiliations:** 1 Division of Epidemiology, Analysis, and Library Services, Center for Surveillance, Epidemiology, and Laboratory Services; 2 Division of Vital Statistics, National Center for Health Statistics; 3 Division of Nutrition, Physical Activity, and Obesity, National Center for Chronic Disease Prevention and Health Promotion; 4 Office of the Associate Director for Science, CDC

In 2010, the top five causes of death in the United States were 1) diseases of the heart, 2) cancer, 3) chronic lower respiratory diseases, 4) cerebrovascular diseases (stroke), and 5) unintentional injuries (1). The rates of death from each cause vary greatly across the 50 states and the District of Columbia (2). An understanding of state differences in death rates for the leading causes might help state health officials establish disease prevention goals, priorities, and strategies. States with lower death rates can be used as benchmarks for setting achievable goals and calculating the number of deaths that might be prevented in states with higher rates. To determine the number of premature annual deaths for the five leading causes of death that potentially could be prevented (“potentially preventable deaths”), CDC analyzed National Vital Statistics System mortality data from 2008–2010. The number of annual potentially preventable deaths per state before age 80 years was determined by comparing the number of expected deaths (based on average death rates for the three states with the lowest rates for each cause) with the number of observed deaths. The results of this analysis indicate that, when considered separately, 91,757 deaths from diseases of the heart, 84,443 from cancer, 28,831 from chronic lower respiratory diseases, 16,973 from cerebrovascular diseases (stroke), and 36,836 from unintentional injuries potentially could be prevented each year. In addition, states in the Southeast had the highest number of potentially preventable deaths for each of the five leading causes. The findings provide disease-specific targets that states can use to measure their progress in preventing the leading causes of deaths in their populations.

Mortality data from the National Vital Statistics System for the period 2008–2010 were analyzed. Population estimates for the period 2008–2010 were produced by the U.S. Census Bureau in collaboration with the National Center for Health Statistics. The calculations of potentially preventable deaths were restricted to U.S. residents and to deaths that occurred to persons aged <80 years. The age restriction is consistent with average life expectancy for the total U.S. population, which was nearly 79 years in 2010 (2). Analysis was restricted to deaths with an underlying cause of death among the five leading causes, based on *International Classification of Diseases, 10th Revision* (ICD-10) codes: diseases of the heart codes (I00–I09, I11, I13, I20–I51), cancer (C00–C97), chronic lower respiratory diseases (J40–J47), cerebrovascular diseases (stroke) (I60–I69), and unintentional injuries (V01–X59, Y85–Y86). The five leading causes of death represented 63% of all deaths in 2010; the next five most frequent causes accounted for only about 12% of deaths (2).

The annual number of potentially preventable deaths for each of the five leading causes of death by state was calculated in three steps. The first step was to calculate and rank state disease-specific death rates by age group. Ages were initially grouped by 10-year increments, from 0–9 years through 70–79 years. However, these 10-year age groups, especially at the younger ages, frequently did not have enough deaths reported to be statistically reliable. Therefore, adjacent 10 year-age groups with small numbers of deaths were combined until enough deaths were aggregated to achieve reliability. For chronic lower respiratory diseases, for example, the age groupings were 0–49, 50–59, 60–69, and 70–79 years. The three states with the lowest observed death rates for each age group-specific cause of death category were then selected and their death rates averaged to calculate a lowest average age-specific death rate for each cause of death. The average of the lowest three states was chosen to minimize the effect of any extreme outlier and to represent the low end of the distribution of death rates among the states. The second step was to calculate expected deaths for each age group and state by multiplying the age-specific state populations by the lowest three-state average age-specific death rate for each cause. Total expected deaths for each cause per state were then calculated by summing expected deaths over all age groups up to age 79 years. Finally, the potentially preventable deaths were calculated by subtracting expected deaths from observed deaths. In instances where the result would be a negative number of potentially preventable deaths because the existing state rate was lower than the average of the three lowest states, the state’s potentially preventable deaths were set to zero. Results are presented by state and by the 10 U.S. Department of Health and Human Services regions.*

During the period from 2008 to 2010, the average number of annual deaths from the five leading causes of death in persons aged <80 years was 895,317. This number represents 66% of annual deaths from all causes. The estimated average number of potentially preventable deaths for the five leading causes of death in persons aged <80 years were 91,757 for diseases of the heart, 84,443 for cancer, 28,831 for chronic lower respiratory diseases, 16,973 for cerebrovascular diseases (stroke), and 36,836 for unintentional injuries (Table 1). The Southeast (Region IV) had the highest number of potentially preventable deaths for all five leading causes of death (Table 2). The proportion of potentially preventable deaths among observed deaths for each of the five causes of death were 34% for diseases of the heart, 21% for cancer, 39% for chronic lower respiratory diseases, 33% for cerebrovascular diseases (stroke), and 39% for unintentional injuries (Figure).

## Discussion

Death rates are population health outcome measures that reflect the combined influences of multiple biological and social health determinants, public health efforts, and medical care. Examining which diseases and injuries result in the greatest number of deaths in populations, particularly for deaths that occur earlier than expected, allows health officials to establish disease prevention goals, priorities, and strategies. In the United States, the largest number of deaths during 2008–2010 occurred from diseases of the heart, cancer, chronic lower respiratory diseases, cerebrovascular diseases (stroke), and unintentional injuries (1). The results of this study demonstrate that if all states achieved the lowest observed mortality levels for the five leading causes, when considered separately, as many as 91,757 premature heart disease deaths, 84,443 cancer deaths, 28,831 chronic lower respiratory disease deaths, 16,973 stroke deaths, and 36,836 unintentional injury deaths might be prevented each year. These calculations translate to approximately one in three premature heart disease deaths, one in five premature cancer deaths, two out of five chronic lower respiratory disease deaths, one out of every three stroke deaths, and two out of every five unintentional injury deaths that could be prevented.

Reducing the number of earlier than expected deaths from the leading causes of death requires risk factor reduction, screening, early intervention, and successful treatment of the disease or injury. For the five leading causes of death, the major modifiable risk factors include 1) *diseases of the heart*: tobacco use, high blood pressure, high blood cholesterol, type 2 diabetes, poor diet, being overweight, and lack of physical activity (3); 2) *cancer*: tobacco use, poor diet, lack of physical activity, being overweight, sun exposure, certain hormones, alcohol, some viruses and bacteria, ionizing radiation, and certain chemicals and other substances (4); 3) *chronic lower respiratory diseases*: tobacco smoke, second hand smoke exposure, other indoor air pollutants, outdoor air pollutants, allergens, and occupational agents (5); 4) *cerebrovascular diseases (stroke)*: high blood pressure, high blood cholesterol, heart disease, diabetes, being overweight, tobacco use, alcohol use, and lack of physical activity (6); and 5) *unintentional injuries*: lack of vehicle restraint use, lack of motorcycle helmet use, unsafe consumer products, drug and alcohol use (including prescription drug misuse), exposure to occupational hazards, and unsafe home and community environments (7).

The majority of these risk factors do not occur randomly in populations; they are closely aligned with the social, demographic, environmental, economic, and geographic attributes of the neighborhoods in which people live and work (8). However, the calculation of potentially preventable deaths in this study did not account for differences in the attributes of states that might influence risk factors and ultimately death rates, such as proportion of the population below the poverty level. If health disparities were eliminated, as is called for by *Healthy People 2020* (9), all states should be closer to achieving the lowest possible death rates for the five leading causes of death.

The findings in this report are subject to at least four limitations. First, uncertainty and error in the diagnosis and reporting of cause of death might result in errors in death rate estimations for some causes of death. Second, state affiliation is based on the person’s residency at the time of death. With the exception of unintentional injuries, the factors that led to the resulting cause of death for some persons might have accumulated over a lifetime spent in different geographic locations. Third, the potentially preventable deaths are based on existing levels of state performance for the three states with the lowest death rates for each condition and might underestimate the benefit if these three states made full use of optimal health promotion and disease prevention strategies. Finally, to the extent that natural (i.e., random) variability in state death rates from year to year is responsible for the selection of the three states with the lowest death rates, there will be a tendency to regress to the mean. The method used tends to slightly overestimate the number of potentially preventable deaths. Nevertheless, the random component of the variation in state death rates is minimal and any bias is also minimal.

What is already known on this topic?

The top five causes of death in the United States are diseases of the heart, cancer, chronic lower respiratory diseases, cerebrovascular diseases (stroke), and unintentional injuries. Death rates for these diseases vary widely across the states because of the distribution of health determinants, access and use of health services, and public health efforts.

What is added by this report?

This report demonstrates that if all states could achieve the lowest observed death rates for the five leading causes, when considered separately, as many as 91,757 premature heart disease deaths, 84,443 cancer deaths, 28,831 chronic lower respiratory disease deaths, 16,973 stroke deaths, and 36,836 unintentional injury deaths might be prevented in the United States each year.

What are the implications for public health practice?

State health officials can use the lower death rates as benchmarks to establish disease prevention goals, priorities, and strategies. Reducing the number of earlier than expected deaths from the leading causes of death requires the joint effort of public health and heath-care organizations and personnel to support risk factor prevention and reduction, screening, early intervention, and successful treatment of diseases or injuries.

As a further note of caution, potentially preventable deaths cannot be added across causes of death by state or for the nation as a whole because of competing risks. For example, for a state that has been able to reduce its heart disease mortality, some premature deaths will be prevented altogether, but others will be pushed to different causes of death. A person who avoids death from heart disease might then be exposed to a higher risk for dying from injury or cancer. The result is that there is less variation by state in the death rate for all causes combined than for any particular cause of death.

States can use the disease-specific aspirational goals for potentially preventable deaths presented in this report in several ways. They can identify other states with similar populations but better outcomes and examine what those are doing differently to address the leading causes of death. Although each state has a unique set of factors that determine health outcomes, states might find neighboring states or states within their region as good sources of information on effective policies, programs, and services. The goals can also be used to educate state policymakers and leaders about what is achievable if they were able to match the best state outcomes.

## Figures and Tables

**FIGURE:**
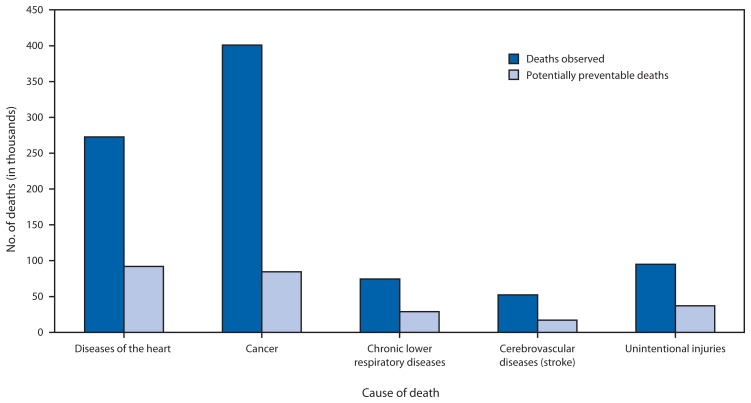
Annual number of deaths observed and potentially preventable* for the five leading cause of death for persons aged <80 years — United States, 2008–2010 * Potentially preventable deaths are observed deaths minus expected deaths (the lowest three-state average age-specific death rate times the age-specific state population) rounded to the nearest whole number.

**TABLE 1:** Annual number of deaths expected,* observed, and potentially preventable† for the five leading cause of death for persons aged <80 years, by state/area — United States, 2008–2010

	Diseases of the heart			Cancer			Chronic lower respiratory diseases			Cerebrovascular diseases (stroke)			Unintentional injuries		
					
State/Area	Deaths observed	Deaths expected	Potentially preventable deaths	Deaths observed	Deaths expected	Potentially preventable deaths	Deaths observed	Deaths expected	Potentially preventable deaths	Deaths observed	Deaths expected	Potentially preventable deaths	Deaths observed	Deaths expected	Potentially preventable deaths
Alabama	6,604	2,993	3,611	7,595	5,227	2,368	1,778	765	1,013	1,277	588	689	2,036	910	1,126
Alaska	463	331	132	703	588	115	112	77	35	91	62	29	331	131	200
Arizona	4,735	3,885	850	7,460	6,775	685	1,558	1,004	554	848	771	77	2,341	1,191	1,150
Arkansas	3,808	1,845	1,963	4,720	3,219	1,501	1,101	476	625	718	365	353	1,221	551	670
California	24,707	19,742	4,965	38,226	34,454	3,772	6,047	4,904	1,143	5,366	3,839	1,527	8,627	6,886	1,741
Colorado	2,815	2,707	108	4,944	4,752	192	1,141	665	476	604	520	84	1,525	940	585
Connecticut	2,569	2,176	393	4,367	3,805	562	509	544	0	425	420	5	905	679	226
Delaware	857	575	282	1,352	1,006	346	224	147	77	170	113	57	296	172	124
DC	729	310	419	742	543	199	73	78	0	107	61	46	169	117	52
Florida	17,586	13,352	4,234	28,249	23,195	5,054	5,327	3,501	1,826	3,481	2,655	826	6,927	3,675	3,252
Georgia	9,103	5,120	3,983	11,820	8,967	2,853	2,413	1,263	1,150	1,965	989	976	3,133	1,791	1,342
Hawaii	1,007	836	171	1,555	1,467	88	141	212	0	244	163	81	344	259	85
Idaho	1,080	883	197	1,753	1,546	207	409	224	185	234	174	60	516	285	231
Illinois	11,424	7,249	4,175	16,558	12,654	3,904	2,740	1,815	925	2,047	1,412	635	3,093	2,395	698
Indiana	6,421	3,783	2,638	9,385	6,612	2,773	2,154	954	1,200	1,240	739	501	2,064	1,209	855
Iowa	2,716	1,892	824	4,127	3,295	832	859	485	374	462	373	89	892	571	321
Kansas	2,248	1,636	612	3,624	2,854	770	826	414	412	485	321	164	1,010	525	485
Kentucky	5,332	2,662	2,670	7,499	4,655	2,844	1,792	675	1,117	934	520	414	2,240	826	1,414
Louisiana	5,784	2,609	3,175	6,909	4,562	2,347	1,106	658	448	1,003	510	493	1,771	850	921
Maine	1,083	928	155	2,259	1,627	632	443	237	206	229	180	49	390	262	128
Maryland	5,321	3,303	2,018	7,218	5,788	1,430	1,035	818	217	935	636	299	1,065	1,093	0
Massachusetts	4,416	3,926	490	8,319	6,865	1,454	1,115	984	131	807	761	46	1,507	1,252	255
Michigan	10,327	6,056	4,271	14,394	10,600	3,794	2,721	1,527	1,194	1,743	1,178	565	2,923	1,869	1,054
Minnesota	2,720	3,050	0	6,273	5,328	945	960	762	198	662	592	70	1,342	993	349
Mississippi	4,183	1,750	2,433	4,731	3,055	1,676	1,016	446	570	827	344	483	1,395	553	842
Missouri	6,553	3,691	2,862	9,023	6,442	2,581	2,090	941	1,149	1,164	724	440	2,328	1,133	1,195
Montana	826	650	176	1,304	1,143	161	341	166	175	162	127	35	416	190	226
Nebraska	1,252	1,063	189	2,254	1,852	402	543	270	273	294	209	85	490	337	153
Nevada	2,903	1,566	1,337	3,370	2,743	627	701	395	306	446	305	141	952	510	442
New Hampshire	916	828	88	1,772	1,455	317	315	206	109	163	158	5	381	255	126
New Jersey	7,106	5,243	1,863	10,948	9,147	1,801	1,436	1,312	124	1,319	1,015	304	1,888	1,665	223
New Mexico	1,510	1,253	257	2,393	2,194	199	535	320	215	310	246	64	1,013	386	627
New York	17,371	11,522	5,849	23,787	20,112	3,675	3,358	2,906	452	2,423	2,246	177	3,804	3,692	112
North Carolina	9,021	5,679	3,342	13,297	9,931	3,366	2,698	1,436	1,262	1,894	1,108	786	3,268	1,802	1,466
North Dakota	512	406	106	780	708	72	170	104	66	127	80	47	193	127	66
Ohio	11,875	7,164	4,711	17,413	12,514	4,899	3,729	1,818	1,911	2,271	1,400	871	4,016	2,184	1,832
Oklahoma	4,857	2,267	2,590	5,787	3,957	1,830	1,736	581	1,155	889	448	441	1,870	703	1,167
Oregon	2,421	2,364	57	5,212	4,153	1,059	1,110	599	511	635	461	174	1,068	730	338
Pennsylvania	12,668	8,221	4,447	19,114	14,340	4,774	3,051	2,101	950	2,194	1,611	583	4,319	2,435	1,884
Rhode Island	820	636	184	1,423	1,112	311	225	160	65	148	123	25	339	200	139
South Carolina	5,413	2,896	2,517	7,063	5,079	1,984	1,391	740	651	1,119	567	552	1,910	883	1,027
South Dakota	590	491	99	1,054	856	198	226	126	100	126	97	29	284	151	133
Tennessee	7,956	3,916	4,040	10,185	6,853	3,332	2,197	995	1,202	1,463	765	698	2,895	1,209	1,686
Texas	19,939	12,683	7,256	27,141	22,143	4,998	5,061	3,139	1,922	4,254	2,471	1,783	7,612	4,551	3,061
Utah	1,229	1,194	35	1,931	2,080	0	383	298	85	282	238	44	765	470	295
Vermont	482	411	71	921	723	198	167	103	64	91	79	12	181	122	59
Virginia	6,588	4,609	1,979	10,162	8,073	2,089	1,647	1,148	499	1,369	891	478	1,889	1,521	368
Washington	4,437	3,844	593	8,193	6,754	1,439	1,451	956	495	907	743	164	1,925	1,269	656
West Virginia	2,400	1,308	1,092	3,415	2,289	1,126	921	338	583	464	257	207	1,031	364	667
Wisconsin	4,513	3,424	1,089	7,530	5,978	1,552	1,190	862	328	869	667	202	1,666	1,074	592
Wyoming	492	333	159	695	585	110	186	83	103	73	65	8	296	106	190
**Total**	**272,688**	**181,261**	**91,757**	**400,949**	**316,652**	**84,443**	**74,458**	**45,738**	**28,831**	**52,360**	**35,390**	**16,973**	**94,862**	**58,055**	**36,836**

**Abbreviation:** DC = District of Columbia.

* Expected deaths are the lowest three-state average age-specific death rate times the age-specific state population rounded to the nearest whole number.

† Potentially preventable deaths are observed deaths minus expected deaths rounded to the nearest whole number.

**TABLE 2:** Annual number of deaths expected,* observed, and potentially preventable† for the five leading cause of death for persons aged <80 years, by U.S. Department of Health and Human Services region§ — United States, 2008–2010

	Diseases of the heart			Cancer			Chronic lower respiratory diseases			Cerebrovascular diseases (stroke)			Unintentional injuries		
					
Region	Deaths observed	Deaths expected	Potentially preventable deaths	Deaths observed	Deaths expected	Potentially preventable deaths	Deaths observed	Deaths expected	Potentially preventable deaths	Deaths observed	Deaths expected	Potentially preventable deaths	Deaths observed	Deaths expected	Potentially preventable deaths
1	10,286	8,904	1,382	19,061	15,587	3,474	2,774	2,234	540	1,863	1,722	141	3,703	2,771	932
2	24,477	16,765	7,712	34,735	29,259	5,476	4,794	4,218	576	3,742	3,261	481	5,692	5,357	335
3	28,563	18,327	10,236	42,003	32,039	9,964	6,951	4,630	2,321	5,239	3,568	1,671	8,769	5,703	3,066
4	65,198	38,367	26,831	90,439	66,962	23,477	18,612	9,820	8,792	12,960	7,538	5,422	23,804	11,650	12,154
5	47,280	30,726	16,554	71,553	53,686	17,867	13,494	7,740	5,754	8,832	5,988	2,844	15,104	9,724	5,380
6	35,898	20,656	15,242	46,950	36,074	10,876	9,539	5,174	4,365	7,174	4,040	3,134	13,487	7,040	6,447
7	12,769	8,281	4,488	19,028	14,443	4,585	4,318	2,111	2,207	2,405	1,628	777	4,720	2,566	2,154
8	6,464	5,782	682	10,708	10,123	585	2,447	1,442	1,005	1,374	1,128	246	3,479	1,985	1,494
9	33,352	26,030	7,322	50,611	45,439	5,172	8,447	6,514	1,933	6,904	5,078	1,826	12,264	8,845	3,419
10	8,401	7,422	979	15,861	13,041	2,820	3,082	1,857	1,225	1,867	1,439	428	3,840	2,414	1,426
**Total**	**272,688**	**181,261**	**91,428**	**400,949**	**316,652**	**84,296**	**74,458**	**45,738**	**28,718**	**52,360**	**35,390**	**16,970**	**94,862**	**58,055**	**36,807**

* Expected deaths are the lowest three-state average age-specific death rate times the age-specific state population rounded to the nearest whole number. Differences between Table 1 and Table 2 are the result of rounding error when calculating states individually or by region.

† Potentially preventable deaths are observed deaths minus expected deaths rounded to the nearest whole number.

§ *Region 1:* Connecticut, Maine, Massachusetts, New Hampshire, Rhode Island, and Vermont. *Region 2:* New Jersey, New York, Puerto Rico, and the U.S. Virgin Islands. *Region 3:* Delaware, the District of Columbia, Maryland, Pennsylvania, Virginia, and West Virginia. *Region 4:* Alabama, Florida, Georgia, Kentucky, Mississippi, North Carolina, South Carolina, and Tennessee. *Region 5:* Illinois, Indiana, Michigan, Minnesota, Ohio, and Wisconsin. *Region 6:* Arkansas, Louisiana, New Mexico, Oklahoma, and Texas. *Region 7:* Iowa, Kansas, Missouri, and Nebraska. *Region 8:* Colorado, Montana, North Dakota, South Dakota, Utah, and Wyoming. *Region 9:* Arizona, California, Hawaii, Nevada, American Samoa, Commonwealth of the Northern Mariana Islands, Federated States of Micronesia, Guam, Marshall Islands, and Republic of Palau. *Region 10:* Alaska, Idaho, Oregon, and Washington. Additional information available at http://www.hhs.gov/about/regionmap.html.

## Footnotes

* *Region 1:* Connecticut, Maine, Massachusetts, New Hampshire, Rhode Island, and Vermont. *Region 2:* New Jersey, New York, Puerto Rico, and the U.S. Virgin Islands. *Region 3:* Delaware, the District of Columbia, Maryland, Pennsylvania, Virginia, and West Virginia. *Region 4:* Alabama, Florida, Georgia, Kentucky, Mississippi, North Carolina, South Carolina, and Tennessee. *Region 5:* Illinois, Indiana, Michigan, Minnesota, Ohio, and Wisconsin. *Region 6:* Arkansas, Louisiana, New Mexico, Oklahoma, and Texas. *Region 7:* Iowa, Kansas, Missouri, and Nebraska. *Region 8:* Colorado, Montana, North Dakota, South Dakota, Utah, and Wyoming. *Region 9:* Arizona, California, Hawaii, Nevada, American Samoa, Commonwealth of the Northern Mariana Islands, Federated States of Micronesia, Guam, Marshall Islands, and Republic of Palau. *Region 10:* Alaska, Idaho, Oregon, and Washington. Additional information available at http://www.hhs.gov/about/regionmap.html.
